# Longitudinal trends in the frequency of medium and fast race winning times in Australian harness racing: Relationships with rules moderating whip use

**DOI:** 10.1371/journal.pone.0184091

**Published:** 2018-03-07

**Authors:** Bethany Wilson, Bidda Jones, Paul McGreevy

**Affiliations:** 1 School of Veterinary Science, University of Sydney, Sydney, New South Wales, Australia; 2 RSPCA Australia, Deakin West ACT Australia; Universidade do Porto Instituto de Biologia Molecular e Celular, PORTUGAL

## Abstract

The use of whips in racing is subject to current debate, not least because the prospect that fatigued horses cannot respond renders the practice futile and inhumane. The racing industries maintain whip use is a form of encouragement and that the rules of racing that govern whip use safeguard horse welfare. The current study examined longitudinal trends in the frequency of medium and fast race winning times in Australian harness racing between September 2007 and August 2016 to explore relationships with a series of changes that moderated whip use. The first change, introduced January 2010, moderated whip action so that horses were struck with less force. Subsequent amendments reversed this change for the final 200m of the race except in one racing jurisdiction. However, those amendments were eventually reversed, restoring the first rule change in all geographic locations. Despite whip use being regulated from January 2010, a long-term trend of increased frequency of both fast and medium winning times over 1609m (~1 mile) was noted. Even after adjusting for this trend, all whip handling codes were associated with greater odds of winning times being less than 1:55 minutes compared with the pre-2010 period. A similar finding for times less than 2:00 minutes did not reach statistical significance. Additionally, the periods immediately before and after introducing the most stringent regulations were compared. This revealed that, when introduced in 2010, these regulations were associated with faster winning times. Their re-introduction in 2016, was associated with no significant differences. Despite concerns that tightening of whip regulations might reduce performance, none of our analyses revealed any significant reduction in either fast or medium winning times in races following the tightening of regulations governing the use of the whip. These findings question the putative need for whips to improve racing performance.

## Introduction

Whip use under various racing codes and the rules that surround it have recently been subject to considerable scrutiny on ethical, welfare [1–11], social sustainability [10], and legal grounds [8]. Proponents of the whip state their confidence in standardised whip design and the rules of racing as means of safeguarding horses against excessive or injurious use of the whip. They argue that “acceptable use of the whip…means that the whip is used for safety (of both jockey and horse) or to encourage the horse to perform to its best when in contention” [12]. Whether the whip can achieve these goals, most notably when horses are so fatigued as to be unable to respond continues to be debated [8]. Meanwhile there is evidence that striking a horse with a padded racing whip would be, at least, aversive and, at worst, possibly painful [4, 13, 14]. An observational study of Australian Thoroughbreds that used high-speed videography found 83% of whip strikes caused indentations of the skin of the horses whipped [4], This is of concern if one considers comparative studies in humans and mice that have demonstrated that such deformation is likely to be detected by cutaneous nociceptors [13], as did a recent study in horses [14].

There has been strong criticism of whip use by some experienced industry observers [15], and RSPCA Australia “is opposed to the use of whips on racehorses for the purpose of enhancing performance as they inflict pain and distress” [16]. Increasingly, there have been calls for the whip to be banned [17].

In harness racing in Australia, whips are traditionally used in training and racing, putatively to encourage horses to give their best performance, to improve race times and to help drivers to control horses. From January 2010, Harness Racing Australia (HRA) began a series of amendments to the whip rules, which are summarised in Table 1.

**Table 1:** Changes to harness racing Australia whip rules from 2009–2016.

Rule period	Duration	Whip Handling Code	Summary of changes	Key wording
Rule period 1	Up to 31 December 2009	A		
Rule period 2	1 January 2010 to 30 September 2010	B	Wording added to require drivers to hold a rein in each hand at all times and only use the whip in a flicking motion or action during a race. www.harness.org.au/rules/Whips-156-156A-121009.pdf	*“A driver shall at all times hold a rein in each hand unless adjusting approved gear”*
Rule period 3	1 October 2010 to 31 March 2011	C (Most states) B (Victoria)	Amendment to allow drivers the option to cross their reins in the final 200 metres of a race. This amendment was not adopted in Victoria. http://www.harness.org.au/news-article.cfm?news_id=14630	*“A driver shall hold a rein in each hand at all times unless he or she is adjusting approved gear* ***or driving in the final 200 metres of a race*.*”***
Rule period 4	1 April 2011 to 31 April 2016	C	Victoria adopts national whip rule. http://www.harness.org.au/news-article.cfm?news_id=15514	
Rule period 5	From 1 May 2016	B	Amendment to allow crossing of reins in last 200 metres of race removed. Additional clauses added to define approved and unapproved whip use. http://www.harness.org.au/rules/AHRR-Whip-Rules-effective-010516.pdf	*“A driver shall hold a rein in each hand at all times unless he or she is adjusting approved gear”*.

From January 2010, the pre-existing whip handling code (Whip Handling Code A–the least stringent across the study period) was replaced with Whip Handling B, the most stringent. Under Whip Handling Code B, drivers were limited in their ability to strike the horse by the necessity of holding a rein in each hand at all times (unless adjusting approved gear) [18, 19]. This allows only a “flicking motion” or motion of the driver’s wrist and elbow, as the hand holding the whip must also hold a rein. The preceding Whip Handling Code A allowed the tip of the whip to be brought back further than the driver’s shoulder.

Whip Handling Code B remained in effect until October 2010 when an exception was introduced for the last 200m of the race in most Australian States [20]. Under this Whip Handling Code C, drivers could cross their reins for the final 200m, allowing more forceful whip strikes during the final stage of a race.

While all other Australian states adopted Whip Handling Code C in October 2010, Victoria, which hosts approximately 25% of harness races in Australia[21, 22], operated under Code B until April 2011 when it too adopted Code C [23].

Code C operated in all Australian states until May 2016, when Whip Handling Code B was again instituted in all Australian states. The rule change was intended to clarify the extant rule and improve “welfare of the animal, public perception and community standards, ethics and the capacity to control the horse”[24, 25].

To summarise, in January 2010, the existing Whip Handling Code A was replaced with two more stringent whip handling codes at various times and locations across Australia. Notably, there were two transitions to the most stringent Whipping Handling Code B from a less stringent code, i.e. from the least stringent Whip Handling Code A in January 2010, and from the interim Whip Handling Code C in May 2016. These two transitions are of particular interest due to potential concerns among some observers that regulating the driver’s use of the whip could result in slower race winning times, and therefore a less exciting product for consumers of Australian harness racing entertainment.

To investigate whether increasing limits on the use of the whip in racing slows horses and thus increases the race times, this report examines the winning times in Australian harness races conducted between from September 2007 to August 2016 for evidence of slower winning times after the introduction of whip rule amendments. It focuses on races over 1609m (~1 mile) in length solely because the industry uses this race length for benchmarking purposes and has done since establishment of the Standardbred breed.

## Data

The data, kindly supplied by Harness Racing Australia (HRA), concerned the winning times of the 133,338 harness races (all of which were at least 1609m (~1 mile) in length) which were administered by HRA members between the 2007–2008 racing season and the 2015–2016 racing season, inclusively. They are summarised in Table 2.

**Table 2:** Winning times recorded in harness racing between the 2007–2008 racing season and the 2015–2016 racing season.

Racing Dates	Whip Handling Code	Slow Winning Times	Medium Winning Times	Fast Winning Times	Rule Period	Months
1 Sep 2007- 31 Aug 2008	A	10,973	2,546	17	1	12
1 Sep 2008- 31 Aug 2009	A	11,897	3,534	29	1	12
1Sep 2009- 31 Dec 2009	A	3,557	1,262	18	1	4
1 Jan 2010- 31 Aug 2010	B	7,763	2,978	73	2	8
1 Sep 2010- 30 Sep 2010	B	666	336	6	2	1
1 Oct 2010- 31 Mar 2011	B (Victoria) C (Other states)	5,336	2,332	95	3	6
1 Apr 2011- 31 Aug 2011	C	4,368	1,930	92	4	5
1 Sep 2011- 31 Aug 2012	C	9,799	5,060	336	4	12
1 Sep 2012- 31 Aug 2013	C	8,612	5,755	538	4	12
1 Sep 2013- 31 Aug 2014	C	7,828	6,421	620	4	12
1 Sep 2014- 31 Aug 2015	C	7,163	6,538	782	4	12
1 Sep 2015- 30. Apr 2016	C	4,207	4,453	806	4	8
1 May 2016- 31 Aug 2016	B	2,037	2,316	422	5	4

Winning times were classified as either slow (> 2:00.1 minutes and slower), medium (between 1:55.1 minutes and 2:00 minutes) or fast (< 1:55 minutes)–see S1 Table. HRA advised that there were no reductions in race lengths programmed over the study period, and therefore any changes in winning times between seasons would not reflect reduced race lengths (personal communication Mr Andrew Kelly, CEO of HRA).

## Analysis

**Fig 1:**
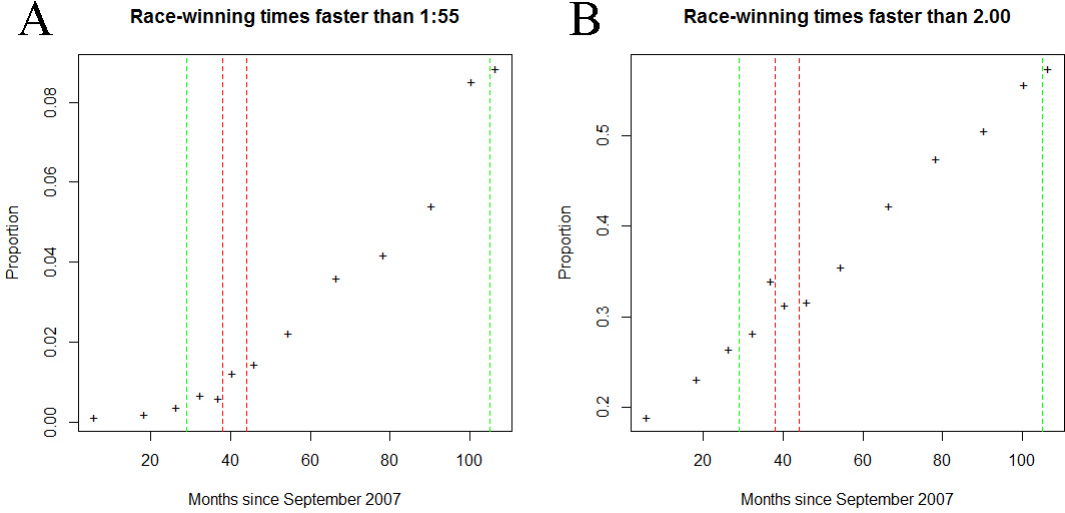
Long-term trend in fast (<1:55 minute) winning times (A) and fast and medium (<2:00 minute) winning times (B) in Australian Harness Racing. Vertical dotted lines denote a change in the whip handling code. Green lines denote a change positive to welfare compared to the preceding period, Red lines denote a change negative to welfare compared to the preceding period.

Preliminary assessment of the data suggested a trend of long-term improvement in race winning times beginning prior to any changes to the whip handling code (Fig 1).

Winning times over the full study period of September 2007 to August 2016 racing were analysed by fitting two logistic regression models, using the glm() function of R:

1. faster or slower than 2:00 minutes (fast and medium winning times vs slow winning times)
2. faster or slower than 1:55 minutes (fast winning times vs slow and medium winning times)

In each case a reduced model, including a variate representing the long -term trends seen in Fig 1 only, was compared to a full model, which also contained a factor representing the whip handling code under which the race took place (either A, B, B and C or C–See Tables 1 and 2). The variate used for the long-term trend was the median month of each period.

Odds ratios were calculated from the logistic regression coefficients, taking Whip Handling Code A as the reference class with an OR = 1, and confidence intervals for each odds ratio were calculated under an assumption of asymptotic normality. The two welfare positive changes to the whip handling code (i.e., the change from Whip Handling Code A to Whip Handling Code B in January 2010, and from Whip Handling Code C to Whip Handling Code B in May 2016) were also scrutinised using a chi-squared test to compare the periods immediately before and immediately after the rule change for evidence that welfare positive whipping code changes resulted in slower race winning times.

## Results

Between 10,724 and 12,562 individual horses raced in races over 1609m in each of the 9 racing seasons included in the data, making 126,295–144,890 starts in the 13,513–15,619 such races, an overall average of 9.2 starters per race.

Between 49.0 to 54.5% of horses that raced in each of the racing seasons recorded at least one winning time, either as an individual winner or in a “dead heat” tie, for a total of 133,501 winning times in the 133,338 races over 1609m over the period.

### Fast winning times compared to slower times

**Fig 2:**
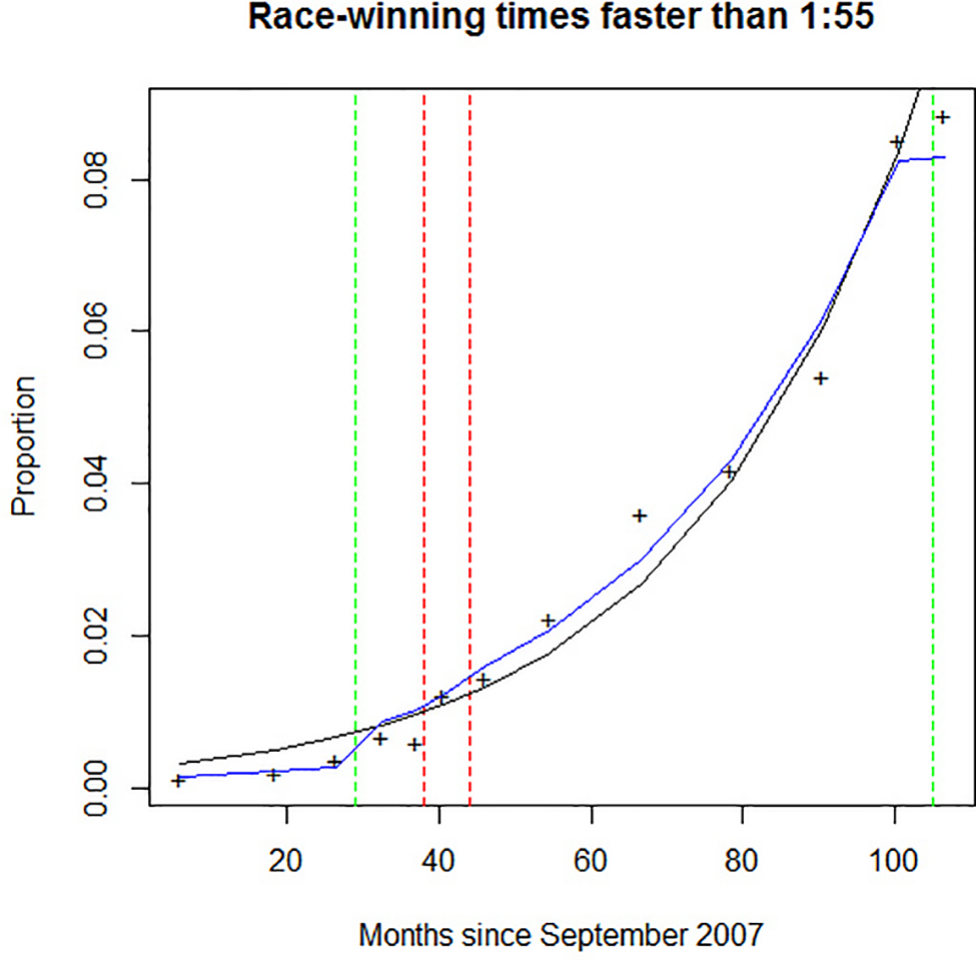
Fit of two logistic regressions model of fast race winning times. The full model included the median month of the time periods considered and the whip handling code of the period (blue line). The reduced model considered only the median month of the time period (black line). Green dotted lines represent changes in whip handling code which are hypothetically positive for welfare; red dotted represent changes in whip handling code which are hypothetically negative with respect to welfare.

A model incorporating both the long-term trend over the study period and the Whipping Rule Code in effect was compared to a model including only the long-term trend over the study period. The goodness-of-fit of the models may be seen in Fig 2.

As illustrated in Fig 2, the full model which included the whip handling codes resulted in a significantly better fit [LR X2 = 108.9, df = 3, p < 0.001] than the model including the long-term trend alone. When compared with (the least stringent) Whip Handling Code A, Whip Handling Code B (OR = 2.75; 95% CI 2.05–3.68), Whip Handling Code C (OR = 3.29; 95% CI 2.50–4.33), and the blend of Whip Handling Codes B and C operating in October 2010-March 2011 (OR = 2.17; 95% CI 2.17–4.12), all had significantly *greater* odds of a fast winning time.

### Fast and medium winning times compared to slow times

**Fig 3:**
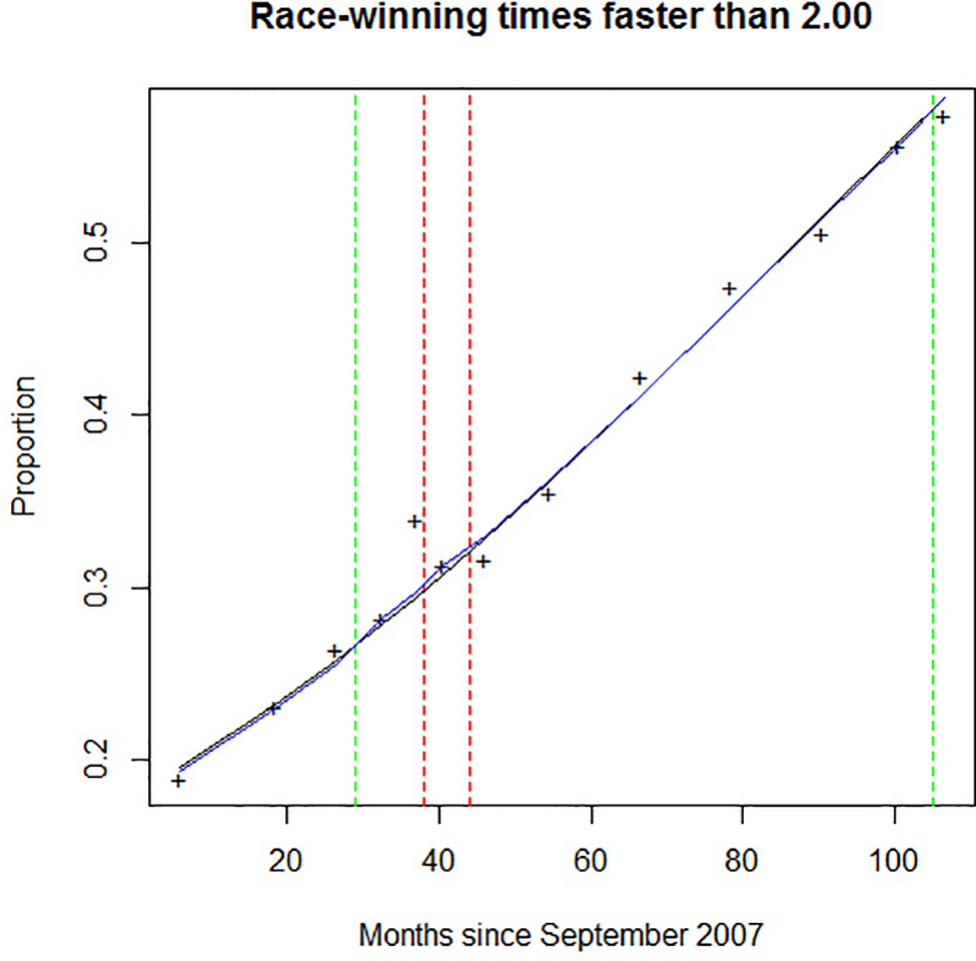
Fit of two logistic regressions model of fast and medium race winning times. The full model included the median month of the periods considered and the whip handling code of the period (blue line). The reduced model considered only the median month of the period (black line). Green dotted lines represent changes in whip handling code which are hypothetically positive for welfare; red dotted represent changes in whip handling code which are hypothetically negative with respect to welfare.

A model incorporating both the long-term trend over the study period and the whipping codes in effect at the time was compared to a model including only the long-term trend over the study period. The goodness-of-fit of the models may be seen in Fig 3.

In contrast to when fast winning times were compared to slower times, when fast and medium winning times were compared to slow winning times, the full model, including the whipping code was not more informative [LR X2 = 3.13, df = 3, p = 0.3718] than the reduced model that included only the long-term trend.

The odds ratios associated with Whip Handling Code B, Whip Handling Code C and the blended period were not significantly different from those associated with Whip Handling Code A.

### October 2010 whip handling code change

In October 2010, a welfare-positive transition from Whip Handling Code A to Whip Handling Code B occurred. A comparison of the frequency of fast, medium and slow winning times during the periods from September-December 2009 and January-August 2010, showed that winning times were significantly different in the later period (*χ*^*2*^ = 9.2767, *df* = 2, *p*-value = 0.009). Specifically, there were *fewer* slow finishing times than expected and *more* medium and fast winning times than expected after the rule change.

However, pairwise post hoc tests did not reveal a significant difference between slow and medium (p = 0.097), slow and fast (p = 0.069) or medium and fast (p = 0.097) winning times before and after the rule change once p values were corrected for multiple comparisons. Nevertheless, there was certainly no evidence of slower winning times after the rule change.

### May 2016 whip handling code change

May 2016 saw the other welfare-positive rule change. In this case, from Whip Handling Code C back to Whip Handling Code B. No significant difference was seen between winning times in the period immediately before and after the change (*χ*^*2*^ = 4.1114, *df* = 2, *p*-value = 0.128).

## Discussion

Despite concerns that tightening of whip regulations might reduce winning times, none of our analyses revealed any significant reduction in either fast or medium winning times in races over 1609m (~1 mile) following the tightening of regulations governing the use of the whip. Analysis of the 2015–2016 Racing Season showed no significant change in the proportion of fast, medium and slow winning times before and after a tightening of regulations and Fig 3 shows no significant effect of whip handling code changes on the proportion of winning times faster than 2:00 minutes.

Indeed, contrary to what might be expected, we found some suggestion of an improvement in the number of winning times faster than 1:55 following the abandonment of (the relatively lenient) Whip Handling Code A in favour of (the more stringent) Whip Handling Codes B and C (see Fig 2 and associated odds ratios) and a suggestion of faster times immediately following the 2010 change to a more welfare-positive rule. However, these findings should be interpreted with care, given the pooling of the data into seasons and the difficulty in separating out the trend of improved winning times over the study period and any effect of the contemporaneous rule changes.

The pivotal difference between Whip Handling Code A and Whip Handling Codes B and C is that the stringent codes (B and C) prevent drivers from using a swinging arm action; approving only flicking actions of the wrist and elbow for most or all of the race. The expectation was that sending the whip through a smaller arc would result in less force on impact. As it happens, in Thoroughbred racing a similar rule requires that jockeys do not raise the whip above the level of their shoulders. It is worth noting that, in Thoroughbred racing, stewards struggle to identify breaches of this rule[4] and the reporting of breaches of this rule varies significantly between metropolitan and country race tracks [26].

Moderation in whip use may avoid horses being put off their stride by being whipped. Interestingly, Evans and McGreevy [3] could find no relationship between whip use and finishing positions in a small sample of Thoroughbred flat races. That finding aligns with those of Deuel and Lawrence who reported that, in Quarter horses, the use of a whip on the shoulder of the leading forelimb, in rhythm with the stride, reduced stride length and increased stride frequency without increasing speed [27].

While track surfaces were not subject to major improvements over the course of the study, a small number of relevant tracks were subject to changes in their circumference, turns and camber. These changes may account for some, or perhaps all, of the improved times at these tracks. Were this the case, the data would still demonstrate that any hypothetical slowing of winning times, after tightening whipping rules, is insubstantial compared to improvements which may be gained by improved track design. From an animal welfare perspective, provided such track modifications do not endanger horses, this is clearly a preferable method of improving times when compared with relaxing whip regulations.

The current study provides evidence that improvements to the welfare of horses participating in harness racing can be made, by restricting the use of the whip as a noxious stimulus, without resulting in slower winning times and, by extension, a less marketable entertainment experience. On the contrary, the reduction or even elimination of whip use may improve the acceptability of harness racing to an increasingly animal welfare conscious public. The current findings raise more questions about the need for the whip as a performance aid in racing and may assist racing authorities in considering the evidence over whether to remove whips from racing altogether. A previous announcement by HRA in December 2017[28], that it would ban the use of the whip, other than for emergency use for safety reasons, was rescinded due to widespread driver and Steward concerns about maintaining safety for drivers and horses[29, 30].

## Supporting information

S1 Table Winning times for horses in races administered by harness racing Australia between the 2007–2008 season and the 2015–2016 season inclusively. See also: http://www.harness.org.au/hra/annual/public/records.htm
(DOCX)
