# Supplementary material for: Longitudinal trends in the frequency of medium and fast race winning times in Australian harness racing: Relationships with rules moderating whip use
Source: PLoS One. 2018 Mar 7;13(3):e0184091. doi: 10.1371/journal.pone.0184091 (PMC5841648; doi:10.1371/journal.pone.0184091)
Supplement: S1 Table — See also: http://www.harness.org.au/hra/annual/public/records.htm (DOCX) [file pone.0184091.s001.docx]

Supplementary 1: Winning times for Horses in Races administered by Harness Racing Australia between the 2007- 2008 season and the 2015-2016 season inclusively.

| **Season** | **Total** | **Ind.** | **Total** | **Total** | **2:00 and faster** | | **2:00.1 and slower** | | **1:55 and faster** | | **1:55.1 and slower** | |
| --- | --- | --- | --- | --- | --- | --- | --- | --- | --- | --- | --- | --- |
|  | Races | **Starters** | **Starts** | **Winners** | Perfs | Ind. Horses | Perfs | Ind Horses | Perfs | Ind Horses | Perfs | Ind Horses |
| 2015-2016 | 14225 | 10724 | 130405 | 5845 | 7997 | 3673 | 6244 | 3808 | 1228 | 740 | 13013 | 5693 |
| 1.09.2015-30.04.2016 | 8605 | n/a | n/a | 4214 | 5259 | 2830 | 4207 | 2818 | 806 | 546 | 8660 | 4459 |
| 1.05.2016-31.08.2016 | 5620 | n/a | n/a | 3151 | 2738 | 1739 | 2037 | 1567 | 422 | 311 | 4353 | 2749 |
| 2014-2015 | 14474 | 11054 | 131707 | 5954 | 7320 | 3432 | 7163 | 4223 | 782 | 487 | 13701 | 5864 |
| 2013-2014 | 14861 | 11377 | 133303 | 5952 | 7041 | 3222 | 7828 | 4476 | 620 | 390 | 14249 | 5887 |
| 2012-2013 | 14909 | 11837 | 138387 | 6052 | 6293 | 3039 | 8612 | 4750 | 538 | 333 | 14367 | 6001 |
| 2011-2012 | 15189 | 12175 | 141714 | 6199 | 5396 | 2693 | 9799 | 5136 | 336 | 222 | 14859 | 6167 |
| 2010-2011 | 15125 | 12496 | 140177 | 6230 | 4791 | 2481 | 10370 | 5346 | 193 | 144 | 14968 | 6215 |
| 1.09.2010-30.09.2010 | 1010 | n/a | n/a | 838 | 342 | 297 | 666 | 582 | 6 | 6 | 1002 | 834 |
| 1.10.2010-31.03.2011 | 7742 | n/a | n/a | 4124 | 2427 | 1513 | 5336 | 3395 | 95 | 79 | 7668 | 4115 |
| 1.04.2011-31.08.2011 | 6373 | n/a | n/a | 3519 | 2022 | 1309 | 4368 | 2810 | 92 | 74 | 6298 | 3498 |
| 2009-2010 | 15619 | 12521 | 144890 | 6430 | 4331 | 2372 | 11320 | 5706 | 91 | 70 | 15560 | 6421 |
| 1.09.2009-31.12.2009 | 4823 | n/a | n/a | 2848 | 1280 | 946 | 3557 | 2355 | 18 | 17 | 4819 | 2843 |
| 1.01.2010-31.08.2010 | 10796 | n/a | n/a | 5228 | 3051 | 1809 | 7763 | 4451 | 73 | 57 | 10741 | 5132 |
| 2008-2009 | 15423 | 12562 | 142429 | 6161 | 3563 | 1962 | 11897 | 5599 | 29 | 26 | 15431 | 6158 |
| 2007-2008* | 13513 | 12022 | 126295 | 5665 | 2563 | 1567 | 10973 | 5288 | 17 | 17 | 13519 | 5660 |

See also: [**http://www.harness.org.au/hra/annual/public/records.htm**](http://www.harness.org.au/hra/annual/public/records.htm)
